# Childhood inflammatory and metabolic disease following exposure to antibiotics in pregnancy, antenatally, intrapartum and neonatally

**DOI:** 10.12688/f1000research.19954.1

**Published:** 2020-02-25

**Authors:** Ronald F. Lamont, Birgitte Møller Luef, Jan Stener Jørgensen

**Affiliations:** 1 Department of Gynecology and Obstetrics, University of Southern Denmark, Institute of Clinical Research, Research Unit of Gynaecology and Obstetrics, Kløvervænget 10, 5000 Odense C, Denmark; 2 Division of Surgery, Northwick Park Institute of Medical Research Campus, University College London, London, HA1 3UJ, UK

**Keywords:** Allergy, Antibiotics, Antimicrobials, Asthma, Atopy, Childhood, Inflammation, Metabolic Disease, Obesity, Pregnancy.

## Abstract

**Background: **There are concerns that the use of antibiotics before, during or immediately after pregnancy may have adverse effects on the neonatal gut microbiome and adversely affect the development of the infant immune system, leading to the development of childhood allergy, asthma, atopic disease and obesity.

**Methods: **In this narrative review, we have explored a number of hypotheses, including the “Barker hypothesis”, the “hygiene hypothesis”, the link between inflammation and metabolic disease, and the influence of the neonatal gut microbiota on the development of the immune system in infants.

**Results:** We found evidence to link the use of antibiotics before, during or immediately after pregnancy with an increased risk of childhood allergy, asthma, atopy and obesity.

**Conclusions** **:** Although we found robust evidence to link antibiotic use in pregnancy with obesity and an “allergic triad” of asthma, eczema and hay fever, care must be taken when interpreting the findings because of the lack of adjustment for confounding variables in published studies. These may be (i) whether or not the mother had the same outcome variable (for example, asthma) as the infant, for which the mother may have received the antibiotics; (ii) the indication, timing or number of antibiotic courses given; (iii) the use of broad-spectrum or narrow-range antibiotics; (iv) the dose-dependent nature of the effector; and (v) the class of antibiotics used.

## Introduction

To establish a link between exposure to antimicrobials before, during and immediately after pregnancy with childhood inflammatory and metabolic diseases such as obesity, asthma and atopic disease, it is necessary to explore a number of hypotheses: (i) the Barker hypothesis, (ii) the hygiene hypothesis, (iii) the link between inflammation and metabolic disease and (iv) the influence of the neonatal gut microbiota on the development of the immune system in infants.

## The Barker hypothesis

The “Barker hypothesis” – in which our health as adults and the quality of life of future generations may be determined by care or complications during fetal or early neonatal life, is now well recognised ^1^. The Romantic poet William Wordsworth, in his 1802 poem, “My Heart Leaps Up”, coined the idiom, “The Child is father of the Man” and this was used in the title of an editorial by Romero to illustrate how the Barker hypothesis pertains to perinatal medicine, which has major implications for obstetricians, neonatologists and society as a whole ^2^. It is becoming increasingly more evident in several studies that insults beyond the obvious examples of genetic or congenital diseases during the earliest stages of life can profoundly affect our health as adults and cause disease. Intrauterine malnutrition is a significant risk factor for later stroke, diabetes, chronic hypertension and death from coronary artery disease in adults. Some causes of male infertility have their origins in inadvertent intrauterine exposure to environmental oestrogens. Under-nutrition during fetal or neonatal life may produce changes in haemostatic factors and in lipid metabolism, which increase the risk of cardiovascular disease. Raised cholesterol levels have been found in adults born with a small abdominal circumference, and increases in fibrinogen and factor VII are found in males born with short stature and a small abdominal circumference, who fail to gain weight during infancy. It is also possible that differences in diabetes, essential hypertension and other adult diseases observed in certain racial groups are not due to a genetic predisposition but are the result of differences in the quality of the intrauterine environment and postnatal life ^2^. A large registry-based study of nearly 2 million Swedish children born between 1973 and 2014 reported the long-term risk of neuropsychiatric disease following exposure to infection *in utero*. The exposure was hospitalisation during pregnancy with a maternal infection. The principal outcome measure was a diagnosis of psychosis, bipolar disorder, depression or autism among offspring. Although there was no evidence of a higher risk of bipolar disorder or psychosis among children exposed to infection *in utero*, fetal exposure to a maternal infection raised the risk of an inpatient diagnosis of autism (hazard ratio [HR] 1.79, 95% confidence interval [CI] 1.34–2.40) or depression (HR 1.24, 95% CI 1.08–1.42) in the child. Although the exposure was hospitalisation during pregnancy with a maternal infection rather than the use of antibiotics *per se*, the inference is that for an infection requiring hospital admission, antimicrobials would have been administered. These findings suggest that fetal exposure to a maternal infection during hospitalisation raised the risk of autism and depression. The findings emphasised how important it is to avoid infections during pregnancy, which may impart subtle fetal brain injuries that contribute to the development of depression and autism ^3^.

## The hygiene hypothesis

In 1989, the results of a cohort of 17,414 British children born during one week in March 1958 and followed up to the age of 23 years were published. Of the 16 social, environmental and perinatal factors addressed, the most striking associations with hay fever (allergic rhinitis) were those for family size and the position in the household during childhood. At the ages of both 11 and 23 years, hay fever was inversely related to the number of children in the household at age 11, an age when it was assumed that most families were complete. Eczema in the first year of life was also independently inversely related to the number of older children in the household. Strachan’s interpretation of the results was that allergic diseases were prevented by infections in early childhood, which were transmitted by unhygienic contact with older siblings or acquired antenatally from a mother infected by contact with her older children. Subsequent infection or reinfection by younger siblings might confer additional protection against allergic disease. Farm-raised children exposed to a variety of bacteria from animals and the soil also have lower rates of asthma. Strachan also hypothesised that, over the previous century, improvements in household amenities, declining family size, and higher standards of personal hygiene had reduced the opportunity for cross-infection in young families. He concluded, “This may have resulted in more widespread clinical expression of atopic disease, emerging earlier in wealthier people, as seems to have occurred for hay fever” ^4^. Although the terminology was not used, the message in this seminal publication has become known as the “hygiene hypothesis” ^4^.

### Acute lymphoblastic leukaemia

In support of the hygiene hypothesis is recent evidence concerning the pathophysiology of acute lymphoblastic leukaemia. Acute lymphoblastic leukaemia is a disease of affluence and the commonest cause of childhood leukaemias. It is also the major cause of childhood cancer in high-income countries ^5^. The incidence of acute lymphoblastic leukaemia is rising by 1% each year in high-income countries and is more common in first-born infants. The theory behind this finding is based on a two-stage gene–environment interaction. The first stage occurs *in utero* with an accidental gene mutation. The second stage occurs postnatally and makes the immune system more prone to react to infection, but only in children brought up in a relatively germ-free environment. According to Hawkes, “the first change loads the gun, the second pulls the trigger” ^5^. In only-children raised in such a sanitised environment with little or no social contact in the first year of life, the development of the immune system is thought to be flawed. This flaw will be less evident in children who are breastfed and those born by vaginal birth. Accordingly, since the immune system evolves in an environment of infections, infection is required for it to form correctly ^6^. Paradoxically, acute lymphoblastic leukaemia is caused by infections but also a lack of infection. The absence of “rough and tumble” in the first year of life leaves the immune system incapable of an appropriate response at a later stage. Although it could be argued that children today are exposed to infection through new vaccination programmes that stimulate the immune system, the antigens used to develop such vaccines are often based on attenuated viruses (as in varicella vaccine) ^7^ or virulence factors such as capsular polysaccharides (as in group B haemolytic streptococcus vaccination) ^8^ rather than the complete, wild organism that may be more antigenic and better able to stimulate an adequate immune response.

## Inflammation and metabolic disease

Among the most basic of defence responses to threat, whether in primitive organisms or in higher-order species, are the responses to a lack of nutrients (starvation) and the capacity to mount an effective immune response to pathogens (attack from a predator). Hence, many immune and metabolic response pathways, or pathogen-sensing and nutrient-sensing systems, alongside the molecules and pathways they comprise, have evolved in parallel and have been evolutionarily conserved throughout species ^9^. The acute response to inflammation is characterised by redness, fever, pain and swelling (Latin: *rubor*, *calor*, *dolor* and *tumor*). This short-term adaptive response is a component of tissue repair and is advantageous to the organism. In contrast, the long-term consequences of prolonged, low-grade or chronic inflammation, that are metabolically triggered by nutrient and metabolic surplus, as in certain metabolic diseases, may not be beneficial and may predispose individuals to obesity and type 2 diabetes ^10^.

### Conceptual considerations and evolutionary perspectives

The response to starvation selects for energy efficiency and favours the storage of excess calories when food is scarce. Conversely, with nutritional surplus, this once-advantageous metabolic state can lead to excess adiposity and its associated problems. The ability to resist infection has also led to Darwinian selection of a strong immune response. This is particularly evident when consideration is given to massive population declines during infectious epidemics and pandemics ^11, 12^. This may be considered a form of “natural selection”. These traits in combination may generate an organism that is highly capable of processing and storing energy and is equipped with a powerful, and potentially hypersensitive, immune response. This relationship between the metabolic and immune response systems and the physiological mechanisms that control these responses in higher organisms has an evolutionary basis from common ancestry.

***Evolutionary perspective.*** An example of this is the fat body of the fruit fly ( *Drosophila melanogaster*), which incorporates the mammalian homologues of adipose tissue and the haematopoietic and immune systems ^13^ and has similar functional and developmental pathways ^9^. Drosophila’s fat body coordinates the appropriate metabolic status and pathogen survival responses. In contrast, in higher organisms, the liver and haematopoietic system and adipose tissue have specialised into discrete functional units or organs but have maintained their developmental heritage, shared with more primitive organisms. Accordingly, the most primitive response systems integrate the pathogen- and nutrient-sensing pathways such that nutrients can induce immune responses and, conversely, pathogens can evoke and regulate metabolic responses.

Taken together, inflammation and metabolic signalling create a delicate balance. Whereas the short-term compensatory and adaptive measures maintain this balance, when one arm overwhelms the other, the outcome is often detrimental in the long term. Accordingly, sustained exposure to pathogens can disrupt systemic metabolic function in both flies and humans. Similarly, chronic malnutrition or over-nutrition can disturb metabolic homeostasis and lead to an aberrant immune response. For those readers who wish to explore this theory further, linking inflammation with metabolic disease, we refer them to the excellent review by Hotamisligil ^9^. For the purpose of our review, we simply want to establish an association between inflammation and metabolic disease which provides a theoretical link between obesity and alterations to the development of the immune system.

### The relationship between antimicrobials given before, during or immediately after pregnancy and childhood obesity

In the past four decades, childhood overweight and obesity prevalence has risen substantially in most high-income countries ^14^. Although the data are scarce, there also appears to be a rapid rise in childhood obesity in low-income and middle-income countries, despite continuing high levels of under-nutrition. Whereas data for children younger than 5 years have appeared in many large country surveys, data about older children and adolescents are less common, and sample sizes tend to be smaller. Nevertheless, comparable surveys show that the prevalence of overweight in these children is also rapidly increasing. A PubMed search indicates that, using the search term “childhood obesity prevention”, the number of published papers rose from about 20 per year in the late 1980s to 60 per year in the late 1990s and to more than 700 in 2018 alone ^15^. At the time the manuscript of this review was submitted, there were already more than 600 such publications listed in 2019.

With an important role in adipogenesis, any alteration in the gut microbiome increases the susceptibility to obesity in later life ^16^. A number of studies have addressed the use of antibiotics in pregnancy and subsequent childhood obesity ^17– 21^, only one of which found no association between antenatal antibiotics and childhood obesity ^21^. A large Danish study indicated that school-age children up to 16 years of age, exposed to antenatal antibiotics, had a nearly 30% rise in the prevalence of obesity ^17^. In another study, antibiotics administered in the second or third trimester, resulted in an 84% greater risk of obesity by 7 years of age when compared with those children not exposed to antenatal antibiotics ^19^. Delivery by caesarean section (CS) compared with vaginal birth was associated with a 46% greater risk of obesity regardless of whether by emergency or elective section ^19^. In a cohort of 527 children, antibiotics (particularly those given in the first and second trimester), but not antifungals, were associated with a raised body mass index by 2 years of age ^20^. Although the weight of published evidence favours an association between the use of antibiotics in pregnancy and childhood obesity, we accept that obesity is a complex phenotype affected *inter alia* by maternal nutrition and health before and during pregnancy. There were also many confounding factors, not fully addressed in the studies cited, such as maternal obesity and diet during pregnancy, gestational diabetes, gestational weight gain and the number of courses of antibiotics administered.

## The influence of the neonatal gut microbiota on the immune system in infants

The human gut is colonised by hundreds of microbial species that provide services (such as the production of enzymes and antitoxins that humans have not needed to evolve for themselves) but that also provide important functions, including development of the host immune system. The use of antibiotics in early infancy has been shown to cause intestinal dysbiosis ^22^, leading to long-term effects on immunological maturation and the potential for chronic disease ^23^. In the human vaginal microbiota, in contrast to the human gut (where increased diversity is considered to indicate eubiosis), increased diversity is associated with dysbiosis, and low diversity of bacteria is considered to be an indication of eubiosis. Low diversity of bacteria has been found in the gut of neonates exposed to antibiotics *in utero*, particularly reduced levels of *Lactobacilli* and *Bifidobacteria*, the primary organisms known to colonise the human gut in infancy and considered to be essential to initiate the immune system.

When used perinatally, antibiotics may disrupt the maternal-to-neonatal transmission of a healthy microbiota from the maternal gut, vagina, skin and breast milk ^24, 25^. In addition to having effects on the microbiome and metabolome, antibiotics may alter epigenetics ^26^ and fetal development ^18^; through these mechanisms, materno-fetal exposure to antibiotics increases the risk of immune-mediated diseases, such as asthma ^26^.

Prolonged rupture of the membranes and intrapartum antibiotics independently lead to a reduction in neonatal transmission of *Lactobacilli* ^27^, and in babies exposed to antibiotics *in utero*, there is delayed colonisation of intestinal microbiota ^28^. The gut microbiota profiles of 198 infants, whose mothers received intrapartum antibiotics, were associated with intestinal dysbiosis at the age of 1 year. This involved a reduction in *Bacteriodetes* and greater quantities of *Enterococcus* and *Clostridium* species, frequently found in association with low gut microbiota diversity in patients with atopic disease. In one study, a degree of “microbiota recovery” throughout the first year of life was reported; this recovery was more evident in infants that were exclusively breastfed ^29^.

To explain this observation, the biological model used involved the disruption of the developing gut microbiota, which resulted in a failure of maturation in the development of the neonatal immune response. This has also been demonstrated in animal studies, where the *in utero* maternal microbiota and metabolites modified the fetal innate immune system and caused allergic airway disease ^30, 31^. The infant immune system is primed *in utero* and modified postnatally. Factors that modify *in utero* microbial exposure may also influence the development of allergy ^32^. Antimicrobials may induce a modification of the neonatal gut microbiome, which affects the developing immune system such that it promotes the development of allergic disease. Epidemiologically, the composition of the gut microbiota in children with atopic disease is different from that of normal controls ^33^.

## The use of antimicrobials before and during pregnancy and in the perinatal period

If we accept the background developed from the “Barker hypothesis” section to the “Inflammation and metabolic disease” section that (i) the normal development of the immune system depends on antigenic exposure and our response to it ^4^, (ii) our adult health and the quality of life for future generations are determined by care or complications during fetal or early neonatal life ^1^ and (iii) the development of our immune response from primitive life to the threat from infection is linked by the same molecules and pathways as our response to the threat of lack of nutrients ^9^, it should not surprise us that the use of antimicrobials immediately before, during and soon after pregnancy may have long-term effects on the development of atopic and metabolic diseases such as asthma, eczema and obesity.

### Extent of use of antimicrobials in pregnancy

Although concerns over teratogenesis from the use of antimicrobials in pregnancy are not major, the use of thalidomide casts a long shadow, so we should not be complacent ^34^. However, antibiotic resistance remains a major problem ^35^. The extent to which antimicrobials in general and antibiotics in particular are used in pregnancy is difficult to quantify as these may be administered in hospital, prescribed by primary care physicians in the community, or self-administered by pregnant women in the form of antifungals or antivirals purchased over the counter to treat self-diagnosed conditions such as herpes labialis, herpes genitalis or vulvovaginal candidiasis. Antimicrobials may be used for infections such as urinary tract infections, which are more common in pregnancy because of the physiological adaptations that are due to progestagenic effects on smooth muscle. Alternatively, they may be used for prophylaxis following preterm prelabour rupture of the membranes or to prevent early-onset neonatal group B streptococcal infection in women at risk of passing this on to the neonate or at the time of CS. A conservative estimate of 40% can be made from a Danish Registry–based study of nearly a million women ^36^ but that is likely to be an underestimate as it applied only to antimicrobial prescriptions filled in the community without data on hospital-based or self-administered antimicrobials.

In a recently published study of 5.6 million pregnancies in the US between 2006 and 2015, 57% of women received antibiotics during hospitalisation for delivery ^37^. The focus of the study was the risk of *Clostridium difficile* infection associated with the use of clindamycin and acute kidney injury associated with gentamicin during hospitalised birth. Although the absolute risk of such complications was low, the receipt of clindamycin was associated with significantly increased likelihood for *C. difficile* infection and receipt of gentamicin with significantly increased likelihood of acute kidney injury. Nevertheless, the impression given was that there was pressure for an increased, not decreased, use of antibiotics during hospitalisation ^37^. This dichotomy may be emphasised by the recommendations for the early use of antibiotics when sepsis is suspected ^38^.

## Evidence linking the use of antimicrobials in pregnancy with allergy, asthma and atopic disease

### Allergy and atopic disease

In 1988, following successive reports from British General Practice Surveys, hay fever was described as a “post–Industrial Revolution epidemic” ^39^. The following year, it was noted that the prevalence of asthma and childhood eczema (allergic dermatitis) had been increasing over the previous 30 years. Currently, atopic disease, a syndrome of hypersensitivity which typically presents as one or more of an “allergic triad” of asthma, eczema and hay fever, affects around 25% of the population, particularly in industrialised countries and especially in children and young adults ^40^. The use of antenatal or intrapartum antibiotics is significantly associated with atopic dermatitis (eczema) in infancy if they are used for more than 24 hours ^41^ or if the infant was delivered by CS ^42^. In the first year of life, cow’s milk allergy (lactose intolerance) accounts for up to 6% of food allergies ^43^. Food intolerance is probably due to a mixture of genetic and environmental factors but may be due to modifiable risk factors such as the use of antibiotics in pregnancy. Two studies reported that the use of antibiotics before or during pregnancy led to an increased risk of lactose intolerance in offspring, although this was less in children who were breastfed for longer periods of time ^44, 45^.

### Asthma

Over the last four decades, the global incidence of asthma has increased; it is estimated that there were 334 million cases in 2014 ^46, 47^. Asthma is also the most common chronic disease in children between 5 and 14 years of age ^46^. Among the risk factors for asthma in offspring are maternal asthma, exposure to cigarette smoke, and air pollution in pregnancy ^48, 49^, together with low birth weight and preterm birth ^50^. Even after controlled for confounding factors, an increasing number of studies have concluded that there is a positive association between childhood asthma and the use of antibiotics in or around pregnancy ^51– 56^. Vitamin D (the “antibiotic vitamin”) is an immunomodulator that is associated with a number of outcomes in pregnancy ^57^ and extra-skeletal outcomes in children ^58^. Supplementation with vitamin D in pregnancy reduces the risk of infection and the need to use antibiotics in pregnancy and also reduces recurrent wheeze and asthma in offspring in childhood at the age of three years ^59^ though not at the age of six. However, care should be taken while interpreting such data. Two large registry-based studies ^55, 56^ suggested that the association between maternal antibiotic use and asthma might be due to a maternal propensity for infection rather than the use of antibiotics *per se*. The association was also stronger when antibiotics were prescribed for respiratory infections. A large population-based cohort study of mother–child pairs ^60^ supported the contention that the association between maternal antibiotic use and asthma was not unique to use during pregnancy. However, it did provide evidence that maternal antibiotic exposure in a dose-dependent manner was associated with an increase in the risk of childhood asthma. After adjusting for infant gender, mode of delivery and method of infant feeding, most classes of antibiotics were associated with an increased risk of asthma, although the degree of risk varied.

## Interventions to reduce adverse effects on the neonatal gut microbiome

### The Neomune Project

The Neomune Project was developed by the University of Copenhagen to study the role of diet and early microbial colonisation in the development of the neonatal immune system and cognitive function in vulnerable preterm or small-for-gestational-age infants. One aim of the Neomune Project is to investigate dietary and microbial elements that help maturation of the immune system in order to develop formula milk to support infants for whom breastfeeding cannot occur or is contraindicated. Another aim is to supplement the limited research on the use of probiotics in milk products. It is thought that these probiotic supplements have the potential to support the neonatal gut microbiome and to improve development of the immune system while suppressing pathogens in vulnerable neonates. For more information, see https://food.ku.dk/english/research_at_food/research-projects/2014/neomune/.

### Seeding therapy

First proposed in 2016, vaginal seeding (sometimes referred to as microbirthing) involves the postnatal transfer of maternal vaginal fluid to neonates born by CS, to correct the disruption of the neonatal microbiota that occurs with CS compared with vaginal birth ^61^. The evidence to provide confidence that vaginal seeding is safe in humans is not robust ^62^, and there is a risk of neonatal transfer of asymptomatic vaginal pathogens such as herpes simplex virus, *Neisseria gonorrhoeae* and *Chlamydia trachomatis.* Since intrapartum antibiotic chemoprophylaxis for the prevention of early-onset group B streptococcal infection of the neonate in the UK and many other European countries is risk-based rather than screening-based ^8^ (as occurs in the USA), there are concerns that this significant cause of neonatal sepsis could be inadvertently transferred to the neonate by vaginal seeding. In the UK and Denmark, obstetricians advise against vaginal seeding and are required to inform women of the potential risks if they intend to pursue this route independently. The introduction of probiotics and breastfeeding are promoted by neonatologists. Ideally, new information about the species-specific function of *Lactobacilli* from the vaginal microbiome project ^63^ will yield additional data for the development of improved probiotics ^64, 65^.

### Breastfeeding

As the first type of food that is introduced into the infant gut neonatally, breast milk is thought to modify the developing microbiota, and, in babies breastfed within the first hour of life, there are similarities between the bacterial constituents of maternal colostrum and new-born meconium ^66^. Species of *Bifidobacteria* are dominant in human breast milk, protect the infant gut from pathogens, and provide health benefits later in life. However, numbers of these bacteria are reduced in the breast milk of women who deliver preterm and in those born by CS, although whether this relates to prophylactic antibiotic cover is unclear ^67^. Accordingly, the American Academy of Pediatrics and the Royal College of Paediatrics and Child Health highlight the importance of breastfeeding and advocate the use of donor breast milk where maternal feeding is contraindicated, or not possible, rather than resorting to the use of formula feeds ^68^.

### Probiotics

The World Health Organization defines probiotics as “live organisms, which when administered in adequate amounts, confer a health benefit on the host” ^69^. Although probiotics purport to balance the gut flora, reliable information is lacking with respect to materno-fetal and neonatal safety. In neonatal mice, T-cell disturbance secondary to the use of antibiotics was prevented by probiotics ^70^, and improved mucosal tolerance following probiotics was reported ^71^. Probiotics may reduce the incidence of pulmonary tuberculosis as well as allergies and asthma in children. However, a higher level of *Bifidobacteria* in infants given probiotics lasted only one week and was associated with an increased risk of infections in later life ^72^. Accordingly, further well-conducted research is required; this will be covered as part of a separate review. Although increased diversity of the gut microbiota is considered a beneficial attribute, the opposite is true of the vaginal microbiota. New information about the vaginal microbiome is providing data about species-specific function of eubiotic, lactic acid–producing organisms that may lead to better probiotic therapies ^63^, but the microbiome of the gut and the vagina are sufficiently complex that the use of a single probiotic, or even two probiotics, may not be able to address this complexity.

## Conclusions

Increasing evidence links the use of antibiotics in pregnancy to subsequent childhood obesity, atopic disease and asthma. This effect appears to be long-lasting and so may also apply to pre-pregnancy and postnatal antibiotic use. Vitamin D, breastfeeding and vaginal birth may have protective effects. Interventions can include alterations to infant diet, breastfeeding or use of probiotics (or a combination of these). However, before “vaginal seeding” can be fully supported, more research is needed. Future research should include long-term follow-up and must be more robust with respect to correcting for confounding variables. It is uncertain whether the mother had the same purported outcome variable as the infant, such as asthma, for which the mother may have received antibiotics. In many studies, there is also a lack of control for the number of antibiotic courses administered, the timing of antibiotic use, whether broad-spectrum or narrow-range antibiotics were used, the indication for antibiotic use and the class of antibiotics used. Greater effort should be made to reduce the use of antibiotics in pregnancy. However, obstetricians are faced with the dichotomy of (i) pressure to reduce the use of antibiotics because of the problems of resistance ^35^ and (ii) the early use of intravenous antibiotics to reduce the serious morbidity and mortality associated with severe sepsis ^38, 73^.
